# Effect on Platelet Function of Metal-Based Nanoparticles Developed for Medical Applications

**DOI:** 10.3389/fcvm.2019.00139

**Published:** 2019-09-18

**Authors:** Nadhim Kamil Hante, Carlos Medina, Maria Jose Santos-Martinez

**Affiliations:** ^1^The School of Pharmacy and Pharmaceutical Sciences, Trinity Biomedical Sciences Institute, Trinity College Dublin, The University of Dublin, Dublin, Ireland; ^2^College of Pharmacy, University of Kufa, Najaf, Iraq; ^3^School of Medicine, Trinity College Dublin, The University of Dublin, Dublin, Ireland

**Keywords:** platelets, metallic nanoparticles, nanoparticles, activation, aggregation, cytotoxicity, nanoparticles charge, nanoparticles shape

## Abstract

Nanomaterials have been recently introduced as potential diagnostic and therapeutic tools in the medical field. One of the main concerns in relation to the use of nanomaterials in humans is their potential toxicity profile and blood compatibility. In fact, and due to their small size, NPs can translocate into the systemic circulation even after dermal contact, inhalation, or oral ingestion. Once in the blood stream, nanoparticles become in contact with the different components of the blood and can potentially interfere with normal platelet function leading to bleeding or thrombosis. Metallic NPs have been already used for diagnosis and treatment purposes due to their unique characteristics. However, the potential interactions between metallic NPs and platelets has not been widely studied and reported. This review focuses on the factors that can affect platelet activation and aggregation by metal NPs and the nature of such interactions, providing a summary of the effect of various metal NPs on platelet function available in the literature.

## Introduction

Nanoparticles (NPs) are defined as particles which range from 1 to 100 nm in size in at least one dimension (length, width, or depth) (1, 2). Although, the development of nanotechnology is a modern science, NPs have always existed in the natural environment and throughout the ages, humans have been continually exposed to airborne NPs (3). However, with the onset of the industrial revolution and the more recent explosion of interest in NPs for scientific development, the exposure to different types of NPs is on the rise. In fact, the use of engineered nanomaterials from a wide range of compositions and sizes together with the study of their potential hazards is continuously increasing.

This review will focus on the interactions of metallic NPs developed for medical applications with blood platelets. The search has been conducted using PubMed, Scopus, and Google Scholar and relevant full text original and review articles published in English up to April 2019 have been included.

### Factors Associated to Nanoparticle's Toxicity

Since the introduction of nanomedicine to describe the application of nanotechnology to benefit patients by The Royal Society and Royal Academy Engineering in 2004 (4), engineered NPs have become promising tools for the monitoring, diagnosis and treatment of human diseases. Despite the ability of humans to avoid, tolerate, and adapt to naturally occurring NPs, the exposure and accumulation of engineered NPs in our body can lead to plausible side effects (4). Multiple studies have already demonstrated that NPs interactions with biological systems depend on size, shape, charge, and the constituent material of the nanomaterial. In fact, those parameters can have a profound effect on cellular uptake and toxicity (5) and therefore on platelet's function (6–8).

#### Nanoparticles Size

Alveolar clearance by macrophages in the lungs constitutes an important barrier for inhaled NPs. However, the efficacy of this mechanism decreases with NPs size leading to a local increased deposition, greater access of NPs to the circulatory system and to potential cardiovascular side effects (9). It has been previously demonstrated that the degree of cytotoxicity and platelet activation and aggregation is inversely correlated with the NPs size (6, 10–12). Therefore, to dampen their potential toxic effect, while improving their stability, various types of surface coatings have been used (7, 13, 14). Surface modification using polyethylene glycol (PEG) for example, has been successfully employed to improve the platelet compatibility of gold NPs (15).

#### Nanoparticles Shape

The physical dimensions of nanomaterials are also strongly correlated with their potential toxicity profiles when compared with the bulk materials of the same composition (16, 17). For example, although carbon black is non-toxic, inhaled carbon nanotubes can result highly toxic (18). One of the factors that strongly influences NPs toxicity is the NPs shape. However, for a given geometric shape, NPs size largely determines the cellular uptake (19–22). Gratton et al. have demonstrated that nanorods larger than 100 nm exhibit the highest rate of cellular uptake followed by spheres, cylinders, and cubes of similar size (23). In contrast, studies using particles smaller than 100 nm have found that spheres show a greater cellular uptake than nanorods (19, 24). However, little is known about the influence of NPs shape on platelet function. Holzer et al. have shown that carbon-based nanomaterials could induce thrombus formation in rodents independently of their shape (25). In contrast, He et al. have recently demonstrated that cuboidal cyclodextrin frameworks enhanced platelet aggregation when compared with their spherical counterpart (26).

#### Protein Binding

When NPs become in contact with biological fluids, electrostatic, dispersive, and covalent interactions will regulate the adsorption of proteins onto NPs, leading to the formation of a dynamic “protein corona” (27, 28). Binding of plasma proteins induce changes in the biophysical properties of the NPs modifying their biocompatibility. In fact, the nature and the concentration of the proteins adsorbed on the NPs can affect their bio uptake, biodistribution, and potential side effects (29–34). The adsorption of some types of plasma proteins may result in an inflammatory response and platelet activation and aggregation. For instance, carbon nanotubes have been reported to bind complement proteins leading to complement activation via both classical and alternative pathways. In contrast, adsorption of complement proteins on the surface of gold colloids has not been associated with complement activation (35–37). The ability of PEG-coatings to protect against platelet aggregation corroborates the hypothesis that physical barriers around NPs may also contribute to the loss of their pro-aggregatory effect (15, 38). However, although the adsorption of fibrinogen onto NPs can contribute to platelet adhesion and initiate thrombogenesis, it has been also demonstrated that the composition of the protein corona on PEGylated NPs doesn't predict their hemocompatibility (39).

#### Nanoparticle Charge

NPs charge plays also an important role in NP-plasma protein interactions (29, 40) and therefore in the blood compatibility of NPs. Positively charge NPs can potentially interact with the negatively charged platelet surface and induce platelet aggregation (41, 42). However, Love et al. found that gold NPs with the same size and opposite charge did not induced platelet aggregation (43). Marginal variation in charge may contribute to a significant difference in protein binding to the NP surface and that may be the case with albumin binding to colloidal gold NPs (37).

## Metallic Nanoparticles and Their Applications in Medicine

Metallic NPs can be easily synthesized and modified with various chemical functional groups which allow them to be conjugated with antibodies, ligands, and drugs. Therefore, the potential applications of metallic NPs in a variety of health-related applications over conventional pharmaceuticals, including targeted drug delivery systems, vehicles for gene and drug delivery, and imaging has dramatically increased (44).

Medical diagnostic applications of metal NPs are plentiful, owing to their ability to interact with external stimuli, including infra-red radiation, ultrasonic waves, and magnetic fields (45–48). Paramagnetic and superparamagnetic NPs have shown a great potential for cancer detection, as evidenced, for example, by the superior ability of iron oxide NPs to detect liver metastases and metastatic lymph nodes (49, 50). Metallic NPs can be also used as therapeutics agents. The antibacterial nature of silver has been already well-established and gold NPs have been found to induce cell toxicity when delivered orally. In fact, silver and gold NPs have been demonstrated to be ideal candidates for the treatment of both, multi drug resistant infections and cancer (51–55). In addition, gold NPs have also shown to exert some anti-angiogenic effect by inhibiting the VEGF activity in collagen-induced arthritis in rats and in human umbilical vein endothelial cells (56–58). The use of multifunctional NPs that integrate diagnostic and therapeutic functions in the same system (theranostic) has been gaining increasing momentum in the nanomedical research and development (R&D). For the management of patients suffering from cancer, for example, nanotheranostic platforms could comprise advanced diagnostics, hyperthermia treatment, and targeted delivery of anticancer drugs. Actually, magnetic iron oxide NPs constitute a good example of such “multitasking” platforms (59).

## Metallic Nanoparticles-Platelets Interactions

In 1977, Berry et al. described for the first time the translocation of nano-sized particles across the alveolar epithelium following intratracheal instillations of 30 nm gold particles in rats. They found large amounts of these particles in platelets of pulmonary capillaries and assumed that there might be a pre-disposing factor for platelet aggregation and microthrombi formation even when those NPs are coated with a biocompatible material (60). In fact, NPs which are not intended for systemic use can, due to their ability to cross epithelial barriers, reach the systemic circulation and interfere with physiological platelet function increasing the risk of cardiovascular disease and vascular thrombosis (61–63). Although, some NPs have been developed for therapeutic purposes aimed to target the injured vascular site mimicking platelet function (64) or to enhance blood clotting (65), the potential unwanted, pro- and/or anti-aggregating, properties of NPs are of significant concern in the field of nanomedicine and may impede the progression of promising engineered NPs to the clinical setting.

Induction of platelet aggregation by metallic NPs has been found by multiple research groups. However, some studies also indicate that metal NPs can inhibit or not affect platelet function (Table 1). The extent to which NPs induce platelet aggregation may depend on multiple NPs factors, as explained in the previous sections, but also on the physiological state of platelets prior exposure to NPs. When platelets are in resting state most metallic NPs seem to be inert (7) becoming more sensitive to NPs in the presence of a threshold shear force or “pre-activation” by critical concentrations of ADP (10, 66, 85). However, non-metallic NPs such as carbon nano-tubes or polymer-based NPs seem to induce platelet aggregation in the absence of any “pre-activating factor” (42, 62).

**Table 1:** Metallic nanoparticles-platelets interactions.

**Composition**	**Size (nm)**	**Charge**	**Concentration tested**	**Nanoparticle shape**	**Effect on platelet function**	**References**
Copper oxide	10	Negative	1.6 × 10^19^ atoms/mL	Rods and spheres	No effect	(66)
Gold	13,20,29	Negative	50 μM	Spheres	No effect	(67)
Gold	5–30	Negative	5–40 μM	Spheres	No effect	(68)
Gold	>60	Negative	5–40 μM	Spheres	Inhibition of ADP induced platelet aggregation	(68)
Gold	20	Negative	5–40 μM	Spheres	Platelet Aggregation	(68)
Gold	40	Negative	5–40 μM	Spheres	Platelet aggregation	(68)
Gold	30	Negative	0.45 mg/mL	Spheres	Inhibition of collagen induced platelet aggregation	(37)
Gold	50	Negative	0.420 mg/mL	Spheres	Inhibition of collagen-induced platelet aggregation	(37)
Gold	18	Negative	5 μg/mL	Spheres	Platelet aggregation	(69)
Gold	9.509 ± 0.56	Negative	2.144 × 10^21^ atoms/mL	Rods and spheres	Platelet aggregation	(66)
Gold	30	Negative and positive	5, 15, 25, and 50 μg/mL	Spheres	No effect	(43)
Gold (PEI-)	20	Negative	50 ppm	Spheres	Platelet aggregation	(70)
Gold (PVP-)	50	Negative	50 ppm	Spheres	Platelet aggregation	(70)
Iron	10–50	Negative	2 × 10^19^ atoms/mL	Rods and spheres	Platelet aggregation	(66)
Iron	10	Not specified	5 mg/mL	Spheres	Inhibition of PMA and arachidonic acid induced platelet aggregation	(71)
Iron oxide (Fe_2_O_3_)	55–65	Not specified-	50 μg/mL	Rods	Platelet aggregation	(72)
Iron oxide (Ferucarbotran)	90	Negative	0.5 mM	Spheres	No effect	(73)
Iron oxide (Ferucarbotran)	90	Negative	10 mM	Spheres	Platelet aggregation	(73)
Iron oxide (Ferucarbotran)	90	Negative	5 mM	Spheres	No effect	(73)
Iron oxide (QD-labeled Ferucarbotran)	90	Negative	0.5 mM	Spheres	No effect	(73)
Iron carbide (PEG)	30	Neutral	1 mg/mL	Spheres	No effect	(74)
Iron oxide (Fe_3_O_4_)	20–30	Not specified-	50 μg/ml	Spheres	Platelet aggregation	(72, 75)
Iron oxide (Ferucarbotran (HSA-)	90	Negative	0.5 mM	Spheres	No effect	(73)
Nickel	62	Neutral	50 μg/ml	Spheres	Platelet activation	(72)
Silver	10–15	Positive	50 μM	Spheres	Inhibition of thrombin- induced platelet aggregation	(67)
Silver	20	Neutral	0.05–0.1 mg/kg i.v. or 5–10 mg/kg	Spheres	Platelet aggregation	(76)
Silver	16–71	Positive	50 μg/ml	Spheres	No effect	(77)
Silver (PVP)	90–240	Neutral	50 μg/mL	Spheres	Platelet activation	(72)
Silver	12	Positive	200 mg/L	Spheres	Platelet activation	(78)
Silver (PEG)	20	Neutral	125–625 μM	Spheres	Inhibition of fibrinogen induced platelet aggregation	(79)
Silver (Colloidal)	10–100	Positive	<10 μg/L	Spheres	No effect	(80)
Silver (citrate coated)	24.3 ± 4.5	Negative	~500 μg/mL	Spheres	No effect	(81)
Silver (PVP coated)	21.6 ± 4.8	Negative	~500 μg/mL	Spheres	No effect	(81)
Titanium dioxide (anastase)	101	Negative	1 mg/kg	Not specified	Platelet aggregation	(82)
Titanium dioxide (rutile)	104	Negative	1 mg/kg	Not specified	No effect	(82)
Titanium dioxide	10 × 40	Not specified-	0.4–10 μg/mL	Rods	No effect	(83)
Titanium dioxide	600–4,000	Negative	10 μg/mL	Spheres	Platelet activation	(84)
Titanium dioxide	20–160	Not specified-	50 μg/mL	Rod	No effect	(72)
Zinc oxide	100–1,200	Negative	10 μg/mL	Rod	Platelet aggregation	(84)
Zinc oxide	150	Negative	1 mg/kg	Not specified	No effect	(82)

*HAS, Human serum albumin; PEG, polyethylene glycol; PEI, polyethyleneimine; PVP, polyvinylpyrrolidone; PMA, Phorbol-myristate-acetate; QD, Quantum dots*.

The exact molecular mechanisms by which metallic NPs influence platelet function is not well-understood. However, their effect on platelet granules and integrin receptors seems to be crucial during the process. Metallic NP-induced platelet aggregation is inhibited when ADP pathway is blocked by an appropriate concentration of clopidogrel or apyrase inhibiting therefore the secondary wave of platelet aggregation which depends mainly on granule release. GPIIbIIIa seems to play also an important role in metallic NP-induced platelet aggregation as in the presence of Arg-Gly-Asp-Ser (RGDS), a tetra-peptide which binds and inhibits activated but not resting GpIIbIIIa, platelet aggregation is attenuated (86).

### Silver NPs

The broad-spectrum antimicrobial properties of silver NPs are well-documented. Their widespread use in both commercial and biomedical applications, due to their large surface-area-to-volume ratio which increases their efficacy against bacteria in comparison to common antibiotics, has raised them to the status of the most commercialized NPs.

The blood compatibility of silver NPs, remains controversial, with several studies that report contradictory results. Spherical silver NPs (10–100 nm) have been found to induce platelet aggregation by increasing intraplatelet Ca^2+^ levels, upregulating GPIIb/IIIa and P-selectin and serotonin secretion (76, 78). Laloy et al. reported that silver NPs increased platelet adhesion but did not exert any further effect on platelet aggregation (77). Coating with PEG inhibits platelet aggregation induced by collagen, ADP, thrombin, and arachidonic acid in a concentration dependent manner (79). Silver NPs spherical in shape, 10–15 nm in diameter and monodispersed, have also been shown to have antiplatelet properties (67). Some variability between studies can be due to the use of different dispersing media (87–89) and Deb et al. have argued that the inhibition of platelet function by silver NPs may be due to the presence of the citric acid used for coating their surface (7). In addition, most of the studies use light transmission aggregometry (LTA) for measuring platelet aggregation and LTA has some limitations: (1) it may not be sensitive enough for studying NP-induced platelet aggregation (90). (2) As silver NPs have light absorbance properties, which are significant from 10 μg/mL, the use of concentrations over this threshold can have a profound effect on the results obtained (77). On the other hand, Smock et al. performed a prospective, placebo controlled in healthy human volunteers to assess the effect of commercially available oral colloidal silver NPs on platelet aggregation *ex vivo* using LTA. They found that platelet activation was not enhanced at peak silver serum concentrations (<10 μg/mL) (80).

### Gold NPs

Colloidal gold was first described as a novel NP vector for tumor directed drug delivery back to 2004 (91). Gold NPs are considered to be one of the safest and most attractive drug-delivery agents due of their inert, non-toxic, and highly permeable properties (92). Still, there are some studies that document toxicity of gold NPs depending on the physical dimensions, surface chemistry, and shape of the NPs studied (19, 20, 93–97).

The compatibility of gold NPs with blood components and their effect on platelet function is not well-established. Although, gold NPs >60 nm in size have no effect on platelet function, smaller NPs (20 nm) can activate platelets. Those NPs could trigger platelet aggregation by a molecular mechanism which involves the platelet canalicular system and the activation of intracellular signaling mechanisms that comprise tyrosine phosphorylation, alpha granule release and P-selectin translocation (10, 98). On the other hand, it has been shown that spherical gold NPs of ~30 nm do not affect platelet aggregation, probably due to the total surface contact and/or the formation of the protein corona (37, 43).

Santos-Martinez et al. have investigated the effect that different NPs may exert on platelet function under flow conditions using a Quartz Crystal Microbalance with Dissipation (QCM-D). The group has demonstrated that QCM-D is more sensitive than the traditional LTA when investigating NP-platelet interactions (69, 99) and found that PEGylation of gold NPs improves their platelet compatibility (15). However, the use of polyethyleneimine (PEI) and polyvinylpyrrolidone (PVP) conjugated NPs can induce platelet activation as revealed by the formation of numerous filopodia and degranulation in equine platelets (70). This effect may be related to the adsorption of fibrinogen onto the NPs surface (5) and could be even more obvious if polyacrylic acid (PAA)-conjugated gold nanoparticles are exposed to platelets as PAA binds to and induce conformational changes of fibrinogen (100) that could potentially have a greater impact on the hemocompatibility of those NPs.

### Iron Oxide NPs

Iron oxide NPs have been extensively used as contrast agents. With the introduction of theranostic systems their use has become more attractive as a novel approach for cancer therapy. The use of those NPs loaded with cytotoxic drugs and functionalized to detect and specifically attack malignant cells could potentially reduce significantly both, side effects of cytotoxic drugs and healthy cells damage.

The effect of iron-based NPs on platelet function in the literature is somehow inconsistent. Some iron-based NPs have been found to induce platelet aggregation as demonstrated by morphological changes using scanning electron microscopy (72). Bircher et al. found that carbon-coated iron carbide magnetic NPs incubated with whole blood induced upregulation of GPIIb-IIIa and P-Selectin but this effect was reversed when NPs were PEGylated (74). In another study, the use of dextran-stabilized iron oxide NPs developed for hyperthermia did not affect either platelet function (101). Deb et al. show in their work that starch-stabilized iron oxide NPs do not exert any effect of platelets while citric acid-stabilized iron oxide NPs inhibited platelet aggregation (7).

Platelet labeling can be of great interest for evaluating the influence of different methods on platelet survival when preparing platelet concentrates or when there is a need to distinguishing between donor and recipient platelets. Iron oxide NPs conjugated with quantum dots have been used previously by Aurich et al. as a non-radioactive alternative for platelet labeling. NPs were successfully internalized in platelets but impaired platelet function at the concentrations needed for labeling. However, this effect was abolished when the NPs where functionalized further with human serum albumin (73).

### Nickel NPs

Compared to its elemental state, nickel NPs exhibit exceptional electrochemical properties and show unusual superparamagnetic properties and stability that make them very attractive in the nanotechnology field (102, 103). In addition, nickel NPs have been used in medicine as catalysts in the production of hydrogen nanoparticles (104). Some studies have demonstrated that nickel NPs induce cytotoxic effects *in vitro* (105, 106) and changes in platelet shape (72). However, despite their wide use in industry, their potential toxic effect to humans has not been extensively investigated.

### Zinc Oxide NPs

Zinc oxide NPs are commonly used in nanomedicine. Spherical NPs have been used as anti-cancer and anti-bacterial agents due to their ability to produce reactive oxygen species (ROS) and induce apoptosis (107, 108). In fact, tetrapod NPs have been recently studied for their antibacterial activity (109), antiviral activity (110, 111), and as a vaccine adjuvant (112). Composite types of nanostructures are also synthesized in various forms including zinc oxide quantum dots and zinc oxide nanoclusters as anti-cancer and anti-bacterial agents (113–115).

Zinc oxide NPs prepared in dispersion medium, citrate or glucose solution have been shown to induce human and canine platelet activation (84). On the other hand, systemic administration of zinc oxide NPs to mice did not induce thrombus formation (82). However, studies looking at the blood compatibility of those NPs are once again scarce.

### Copper NPs

Deb et al. have shown that copper NPs have a pro-aggregatory effect activating purinergic receptors (P2Y12) in the presence of ADP at suboptimal concentrations (66). However, Major et al. using a nitric oxide generating polymeric material (polyurethane containing copper NPs) combined with the intravenous infusion of a nitric oxide donor (S-nitroso-N-acetylpenicillamine-SNAP) observed that platelet function was preserved in rabbit blood exposed to extracorporeal circulation (116).

### Titanium Dioxide NPs

Titanium dioxide NPs are widely used in cosmetic products and sunscreens (117) and they are very well-known photocatalysts able to generate different reactive species that could be potentially toxic to microorganisms. In fact, *in vitro* studies have shown deleterious effect of those NPs on different cell lines (118, 119). Titanium dioxide NPs have shown to activate dog and, to a lesser degree, human platelets *in vitro* (84). When administered systemically to rats, titanium dioxide anatase but not titanium dioxide rutile NPs of similar size (around 100 nm) were found to increase murine platelet aggregation (82). In another study, systemic administration of titanium dioxide rutile NPs did not activate platelets or exert prothrombotic effects in mice either (120). However, the intratracheal administration of rutile titanium oxide nanorods has resulted in platelet aggregation in rats (83).

## Conclusions

Medicine is envisaged to be one of the primary beneficiaries of the nanotechnological development. The growth of nanotechnology has fired the “technological boom” in diagnostics, imaging, and drug delivery (121). The scientific community has shown significant interest in further developing the potential medical applications of metal NPs. However, nanomaterial-blood interactions are inevitable regardless of the use NPs are intended to.

The compatibility of NPs with blood elements remains as a controversial topic. Several studies utilizing similar experimental protocols and assessing similar end points report contradictory results and Table 2 summarizes the main effects of metallic NPs on platelets found in the literature. Although there are multiple “NP dependent” factors that can play an important role on NPs-platelets interaction, other circumstances should be kept on mind when carrying out those experiments as they may potentially contribute to the conflicting results: (a) platelet preparation/platelet handling; (b) use of different solutions/dispersing media when preparing the NPs/performing the experiments; (c) presence of impurities or additional substances in metallic NPs or their suspensions; (d) interference of the NPs tested with the method used; (e) use of different concentrations of NPs and or platelet agonists in different experimental settings; and (f) lack of sensibility for detecting platelet microaggregates with the method used.

**Table 2:** Effect of metallic nanoparticles on platelet function.

**Nanoparticle type**	**Main findings**
Silver	Silver NPs need to accumulate within platelets to interfere with different intraplatelet pathways (67, 76, 78, 79). Primary NPs size is as important as the stability and monodispersity of the NPs which highly depend on the type of dispersion medium used. Chitosan and PEG prevent agglomeration (122) but also increase NPs size affecting NPs properties and leading to inconsistent results despite using similar or close primary NPs size. PVP has no effect on platelet function but citric acid can inhibit platelet aggregation (7, 77). Silver NPs can also interfere with light absorbance and therefore, light transmission aggregometry, which is the gold standard for studying platelet function, may not be suitable to investigate silver NPs-platelets interactions
Gold	Gold NPs size is important when investigating their effect on platelet function. It seems that gold NPs >60 nm do not modify platelet function. However, when NPs are <50 nm they can be internalized and accumulated in platelets affecting their function (10, 66, 68–70, 93). PEI, PVP, and PAA coatings can induce platelet aggregation (70, 100, 123)
Iron oxide	The effect of iron nanoparticles on platelet function is not consistent and the studies available demonstrate that they can induce (66, 72, 73, 75); have no effect (73, 74) or inhibit platelet aggregation (71). No single factor seems to be determinant but coatings may play an important role as HAS, PEG, and citrate have shown to do not affect platelet function (73, 74) but PAA inhibited platelet aggregation (71)
Titanium dioxide, zinc oxide, nickel, and copper oxide	Most studies refer to pro-aggregatory effect of these NPs. However, information is scarce to draw a robust conclusion regarding their effect on blood platelets

*PEI, polyethyleneimine; PVP, polyvinylpyrrolidone; PAA, poly acrylic acid; HAS, Human serum albumin; PEG, polyethylene glycol; PMA, Phorbol-myristate-acetate*.

Unwanted side effects of NPs is of significant concern in the field of nanomedicine and often hampers the progression of promising nanomaterials to the clinical setting. Knowledge regarding NPs toxic potential is still limited along with an absence of appropriated regulatory policies for their use and testing (124, 125). In fact, not all commercially available nanomaterials have a Safety Data Sheet and novel nanomaterials are sometimes manipulated without full assessment of their potential health risk (124).

There is no doubt that the development of nanomaterials intended for human use should be always carried out in tandem with an extensive toxicological evaluation. Those studies must include the investigation of the effect of nanomaterials on platelet function as they can lead to secondary effects (bleeding or thrombosis) that can affect individuals involved in their production, manipulation or use during medical applications.

## Author Contributions

NH, MS-M, and CM designed the structure of the different sections of the manuscript. NH did the search and wrote the first draft of the manuscript. All authors contributed to manuscript revision, read, and approved the submitted version.

### Conflict of Interest Statement

The authors declare that the research was conducted in the absence of any commercial or financial relationships that could be construed as a potential conflict of interest.

## Abbreviations

NPs: Nanoparticles
VEGF: Vascular endothelial growth factor
PEG: polyethylene glycol
LTA: light transmission Aggregometry
QCM-D: Quartz Crystal Microbalance with Dissipation, PEI, polyethyleneimine
PVP: polyvinylpyrrolidone
PMA: Phorbol-myristate-acetate
PAA: polyacrylic acid
ROS: reactive oxygen species
SNAP: S-nitroso-N-acetylpenicillamine
HAS: human serum albumin.
